# The Influence of Food Names with Different Levels of Concreteness on Evaluations of Food Deliciousness and Healthiness

**DOI:** 10.3390/foods13162559

**Published:** 2024-08-16

**Authors:** Zhao Yu, Yixin Kang, Peipei Liu, Haokai Ou, Wei Zhang, Xianyou He

**Affiliations:** 1 Key Laboratory of Brain, Cognition and Education Sciences, Ministry of Education; School of Psychology, Center for Studies of Psychological Application, Guangdong Key Laboratory of Mental Health and Cognitive Science, South China Normal University, Guangzhou 510631, Chinaxianyouhe@163.com (X.H.); 2 Key Laboratory of Chinese Learning and International Promotion, South China Normal University, Guangzhou 510631, China

**Keywords:** food names, levels of concreteness, deliciousness, healthiness

## Abstract

Recently, many restaurateurs in the food and beverage industry started using vague and abstract names to label their dishes. However, the influence of the concreteness of food names on consumers’ evaluations of food remains unclear. Therefore, the present study investigated people’s perceptions of food names with different levels of concreteness and their evaluations of food deliciousness and healthiness through two experiments. Experiment 1 investigated the likelihood of names with different levels of concreteness being perceived as foods or dishes through subjective guessing tasks. In line with the hypothesis of mental imagery consistency, the results revealed that individuals were more inclined to perceive high-concreteness names as actual food or dishes than low-concrete names. Experiment 2 further explored the impact of food names with different levels of concreteness on consumers’ perceptions and evaluations of food in terms of the direct sensory (deliciousness) and indirect inference (healthiness) dimension. The results showed that in terms of deliciousness, consistent with the feelings-as-information theory, high-concreteness food names were rated significantly higher than low-concreteness ones. In terms of healthiness, consistent with the incongruence theory, low-concreteness food names were rated significantly higher than high-concreteness ones. These results indicated that high-concreteness names were more likely to be perceived as foods or dishes. Moreover, they also had advantages in the direct sensory dimension (deliciousness) but were perceived as less healthy in the indirect inference dimension (healthiness). The present findings provide new evidence for studies related to food naming and the evaluation of deliciousness and healthiness and offer suggestions and strategies for the food and beverage industry in naming foods and dishes.

## 1. Introduction

In everyday life, we are accustomed to choosing dishes or foods based on their names on the menu. These food names provide consumers with a wealth of information, such as information on ingredients, sensory experiences, geographical characteristics, and flavors [1,2,3]. Therefore, can merely changing the name of a food alter people’s perceptions of its taste and influence consumption?

Consumers rely on branding elements to form expectations and make purchase decisions regarding food [4]. Branding attributes (e.g., brand names, brand logos, brand personality) provide a rich source of information and help consumers choose food [5,6]. Research has suggested that specific brand elements can significantly influence consumers’ perceptions and preferences for the brand or product [7,8,9]. Among these elements, the brand name stands out as particularly important. An increasing body of research has shown that a brand name can influence perceived attributes [10] and purchase intention [11]. Research on food branding has shown that the length of brand names influences the expectations of healthiness in foods and preference for healthy foods. Specifically, foods with shorter (vs. longer) brand names are perceived as healthier, and consumers prefer such foods [12]. Previous research on food selection has shown that the attractiveness of food names and the ease of pronunciation significantly influence consumers’ evaluations of food and their consumption behavior [13,14,15]. For example, Wansink et al. [16] found that people gave a large number of positive evaluations to foods with appealing descriptive names, rating them as more attractive, tastier, and higher in calories than foods with regular names. Another study on plant-rich dishes revealed that attractive dish names (emphasizing ingredients, flavors, etc.) promoted consumer selection of dishes more than those in the control group [17]. Additionally, Kim [18] also found that customers perceived restaurants with sensory food names (e.g., “buttery pasta”) and nostalgic food names (e.g., “mom’s homemade roast chicken”) as offering warmer service than restaurants without such names. In addition to solid foods, names can also impact the consumption of liquids (e.g., drinking water). Cho [19] found that people’s perceptions of water purity and taste change based on the ease of processing brand names. Bottled water with a brand name that is short and easy to pronounce was considered purer. Bottled water with a smoother name was more likely to be considered “better than average”. Thus, adjusting and changing food names seems to be effective and beneficial for food evaluation and sales [20,21].

Existing research has also explored the fundamental classifications of food names. Li [22] categorized the naming characteristics of Chinese cuisine into two main categories: direct naming and associative naming. Direct naming usually includes food’s raw ingredients, taste, color, and cooking methods, such as “手撕鸡 (shǒu sī jī, hand-shredded chicken)”. Associative naming, on the other hand, often incorporates the rich cultural connotations of Chinese dishes, exemplified by dishes such as “佛跳墙 (fó tiào qiáng, Buddha Jumps over the Wall)”, which references a poem. Yan [23] broadly categorized Chinese dish names into two types: realistic and impressionistic. Realistic dish names are mainly based on the dish’s raw materials or cooking methods. Impressionistic names are further divided into two types: one containing positive connotations and the other including people’s names, place names, or certain historical stories. In addition to Chinese cuisine, Miller and Kahn [24] classified Western dish names similarly, categorizing food names into common descriptive names and ambiguous names.

As a result, whether food names are based on descriptive information such as ingredients, cooking methods, or knife skills generally results in specific food names. On the other hand, food names based on cultural connotations or containing positive connotations tend to be more abstract. However, whether food names with different levels of concreteness also affect consumers’ evaluations of food deliciousness and healthiness remains an open issue.

## 2. Theoretical Background

Previous research has also explored the impact of information concreteness on comprehension. Some studies have proposed a concreteness effect, by which that concrete information has an advantage over abstract information in information processing because it is more vivid and detailed and captures more attention [25,26,27]. However, other studies have also reported an abstract effect, by which abstract information exerts a stronger influence on individuals’ information processing and judgment than concrete information [28]. Additionally, some studies have failed to observe a greater impact of concreteness or abstractness on judgment [29]. Research on consumer psychology and marketing psychology has also explored the impact of abstract and concrete information on consumer behavior and purchase decisions. The findings in this area have been mixed. Some studies have found that concrete product descriptions can positively influence people’s decision-making. For example, Balcetis et al. [30] found that when information emphasized the benefits of eating healthy food or the costs of not eating healthy food using clear, detailed language, people were more inclined to choose healthy food over unhealthy ones. However, other studies have shown that abstract descriptions can also positively impact people’s decision-making. Papies et al. [31] found that descriptions emphasizing the embodied simulation or experience of eating and enjoying food increase its attractiveness more than descriptions focusing solely on ingredients.

Similarly, in the field of product consumption and evaluation, there are also two main explanatory theories. Incongruency theory posits that individuals make judgments in the consumption choice process by evaluating the degree of incongruence between new experiences and their existing expectations. When a new experience does not match previous expectations, individuals engage in more effortful or detailed processing to reconcile the incongruence [32]. Generally, the relationship between incongruence and preference is U-shaped [33]. Specifically, moderate incongruence, while requiring more cognitive effort, enables individuals to resolve the incongruence and find meaning in it, making the product appear more interesting and leading to positive evaluations [34].

However, the feelings-as-information theory offers a different perspective. This theory posits that subjective experience plays a role in decision-making and judgment, suggesting that consumers use their feelings (e.g., processing fluency) as sources of information during the judgment process [35]. Related research has found that when consumers interpret verbal information such as dish names, they tend to construct mental images [36] or visualize the food based on the dish name [37]. In most cases, it is more difficult to construct mental images based on ambiguous, abstract names than based on ordinary, concrete descriptive names. Therefore, naming strategies that include specific, direct descriptions of food ingredients or cooking methods usually enable consumers to understand them more easily. In contrast, associative and impressionistic naming strategies that involve cultural allusions may be more challenging for those unfamiliar with the food’s background, making it more difficult to visualize the corresponding food.

In food selection and food decision-making research, deliciousness and healthiness are key attributes [38,39,40]. However, deliciousness and healthiness seem to be distinct attributes. Deliciousness is a more fundamental and hedonic attribute [41,42] that drives behavior through an impulsive system involving automatic, emotional, and rapid evaluations [43]. On the other hand, healthiness is related to the reflective system. Healthiness is a more abstract, less hedonic attribute [41,42,44] that influences the reflective system through conscious and careful control mechanisms to achieve long-term goals [43]. Other studies reinforce this perspective. Research has found that taste is associated with basic, immediate outcomes, while healthiness is associated with abstract, delayed outcomes. Additionally, differences in the speed of decision circuit processing have been observed for basic attributes such as deliciousness and more abstract attributes such as healthiness [45]. Therefore, the evaluation of deliciousness likely falls under the direct perception dimension, while the evaluation of healthiness falls under the indirect inference dimension.

Therefore, this study draws on Miller and Kahn’s [24] classification of food names (common descriptive names and ambiguous names), categorizing food or dish names into two types—high-concreteness and low-concreteness—to investigate the impact of the concreteness level of Chinese food names on the evaluation of food deliciousness and healthiness. The study hypothesizes that because the evaluation of food deliciousness falls under the direct perception dimension, it should follow the basic expectations of the feelings-as-information theory. Specifically, high-concreteness names can better stimulate people’s sensory and emotional experiences, making them more likely to evoke appetite and interest. Therefore, compared to low-concreteness names, high-concreteness names are more likely to be perceived as foods or dishes. Additionally, foods with high-concreteness names will be perceived as more delicious. The evaluation of healthiness, which falls under the indirect inference dimension, should follow the expectations of the incongruency theory. Specifically, for low-concreteness food names, consumers need to exert more cognitive effort to construct mental images and establish connections with the food, which can trigger a high level of curiosity and provide meaning. Therefore, they are likely to positively evaluate the food in terms of healthiness. To test these two basic hypotheses, the present study aimed to investigate people’s perceptions of food with different name concreteness levels and their evaluations of food deliciousness and healthiness through two experiments. Experiment 1 investigated the likelihood of names with different levels of concreteness being perceived as foods or dishes through subjective guessing tasks. Experiment 2 further explored the impact of food names with different levels of concreteness on consumers’ perceptions of food in terms of the direct sensory dimension (deliciousness) and indirect inference dimension (healthiness).

## 3. Pilot Material Ratings

### 3.1. Participants

A total of 34 participants were recruited to participate in the pilot material ratings. All the participants were right-handed with normal or corrected-to-normal vision, were without dyslexia, and were paid for their participation. Four invalid data were excluded due to the failing of attention check or having the same answer for two-thirds of the questions. The remaining 30 valid data, aged between 18 and 25 years (16 males, *M* = 20.57 years, *SD* = 3.63), were used for data analysis.

The experiment was approved by the Institute Ethics Committee, South China Normal University, and written informed consent was obtained from all participants in accordance with the Helsinki Declaration.

### 3.2. Materials

The present study used two categories of materials: food names and food pictures. The 45 food names were selected from 340 regional classic dishes released by the Chinese Cuisine Association and the dishes that appeared in “The Dream of Red Mansions”. There were 23 food names with a high concreteness level (e.g., 松鼠桂鱼, sōng shǔ guì yú, sweet and sour mandarin fish) and 22 food names with a low-concreteness level (e.g., 蚂蚁上树, mǎ yǐ shàng shù, sautéed vermicelli with spicy minced pork).

A total of 45 color photographs of food were selected from the public archives on http://www.baidu.com (accessed on 15 July 2023) and were standardized using WPS Pictures, with all photos being 600 × 450 pixels.

### 3.3. Procedure

Participants were instructed to rate 45 randomly presented food names and 45 food pictures on a 7-point scale, in terms of (i) concreteness of food names (1 = not very concrete; 7 = extremely concrete) using Altarriba’s scale [46], (ii) familiarity of food names (1 = very unfamiliar; 7 = very familiar), and (iii) deliciousness of food images.

### 3.4. Results

According to the ratings, nine food names with a high concreteness level and nine with a low concreteness level were selected as the experimental materials. The paired-sample *t*-test results showed that the group with a high concreteness level (*M* = 4.37, *SD* = 1.07) had a significantly higher concreteness level than the group with a low concreteness level (*M* = 2.51, *SD* = 0.88), *t* (29) = 7.18, *p* < 0.001, Cohen’s *d* = 1.90. The results of the pilot material ratings confirmed that there was no significant difference between familiarity ratings of food names with a high concreteness level (*M* = 2.49, *SD* = 0.81) and those with a low concreteness level (*M* = 2.19, *SD* = 0.72), *t* (29) = 1.97, *p* = 0.058, Cohen’s *d* = 0.39. There was also no significant difference in the deliciousness ratings of food images between those associated with high-concreteness food names (*M* = 5.16, *SD* = 0.60) and low-concreteness food names (*M* = 4.93, *SD* = 0.85), *t* (29) = 1.84, *p* = 0.077, Cohen’s *d* = 0.31 (Table 1).

## 4. Experiment 1

Experiment 1 aimed to investigate whether the concreteness level of food names affect food perception and judgment through subjective guessing tasks.

### 4.1. Participants

A different group of 113 participants were recruited to participate in Experiment 1. All participants were right-handed with normal or corrected-to-normal vision, without dyslexia, and were paid for their participation. Eleven invalid data were excluded due to participants failing the attention check or having the same answer for two-thirds of the questions. The remaining 102 valid data, aged between 18 and 28 years old (53 males, *M* = 21.83 years, *SD* = 1.47), were used for data analysis.

### 4.2. Design

Experiment 1 involved a single factorial design (food names with different levels of concreteness: high-concreteness food names vs. low-concreteness food names). The variables were manipulated within subjects. The dependent variable was the judgment of foods or dishes.

### 4.3. Materials

Based on the results of the material rating experiment, 9 high-concreteness food names (e.g., 糖蒸酥酪, táng zhēng sū lào, sweetened junket) and 9 low-concreteness food names (e.g., 蚂蚁上树, mǎ yǐ shàng shù, sautéed vermicelli with spicy minced pork) were selected as materials for Experiment 1.

### 4.4. Procedure

Participants were randomly presented with 18 food names, without being informed that these were actually the names of foods, and were then asked to rate the likelihood that each name represented foods or dishes on the Lickert Level 7-point scale (1 = completely impossible; 7 = completely likely).

### 4.5. Results

**Figure 1:**
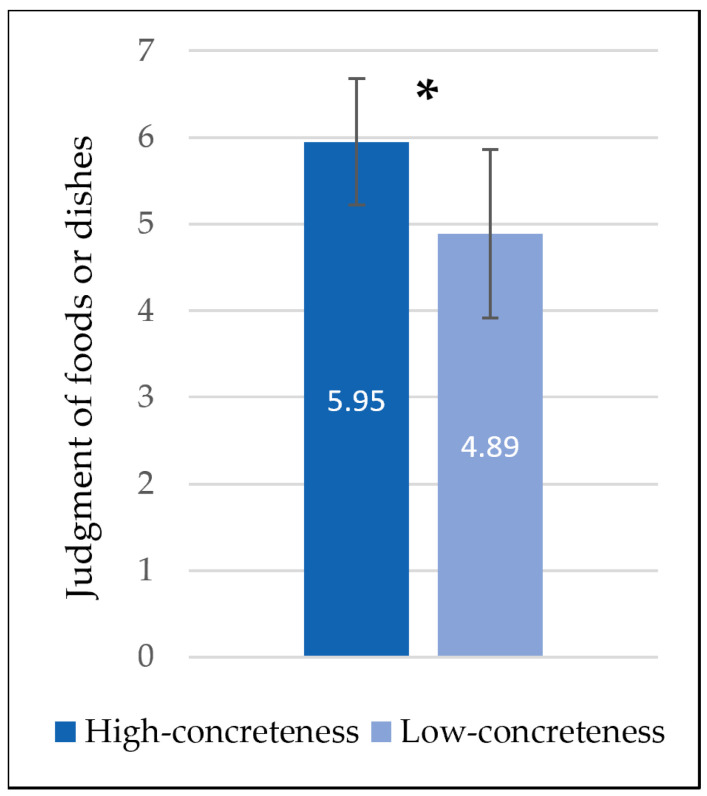
Judgments of the likelihood that food names with different levels of concreteness are perceived as food or dishes. Error lines represent standard deviations. * represents significant difference.

The difference-value (D-value) between the means of the high-concreteness and low-concreteness name groups was calculated, and the normality test was performed. The Kolmogorov–Smirnov (K-S) test revealed that all variables were normally distributed (*p* = 0.200). The paired-sample *t*-test results (Table 2, Figure 1) showed that the high-concreteness name group (*M* = 5.95, *SD* = 0.73) was significantly more likely to be considered as foods or dishes than the low-concreteness name group (*M* = 4.89, *SD* = 0.97), *t* (101) = 11.09, *p* < 0.001, Cohen’s *d* = 1.10.

In line with our hypothesis, the results of Experiment 1 suggest that in the subjective guessing tasks, a high-concreteness name is more likely to be perceived as a food or dish than a low-concreteness name. The reason may be that high-concreteness names contain information about raw materials, taste, color, and cooking methods of food, making it easier for individuals to process and simulate their sensory and emotional experiences. Experiment 2 further predicted that high-concreteness food names have an advantage in the direct perception dimension (deliciousness), whereas low-concreteness food names have an advantage in the indirect inference dimension (healthiness).

## 5. Experiment 2

Experiment 2 aimed to examine the impact of food names with different levels of concreteness on consumers’ perception of food in terms of direct sensory dimension (deliciousness) and indirect inference dimension (healthiness). Experiment 2 focused on examining the participants’ evaluations of deliciousness and healthiness.

### 5.1. Participants

A total of 111 participants were recruited to participate in Experiment 2. All the participants were right-handed with normal or corrected-to-normal vision, were without dyslexia, and were paid for participation. Ten invalid data were excluded due to the failure of attention check or having the same answer for two-thirds of the questions. The remaining 101 valid data were used for data analysis, with ages between 18 and 30 years (52 males, *M* = 21.70 years, *SD* = 1.79).

### 5.2. Design

Experiment 2 involved a 2 (food names with different levels of concreteness: high-concreteness food names vs. low-concreteness food names) × 2 (evaluation dimension: deliciousness vs. healthiness) within-subjects design. The dependent variables were the rating scores of the direct perception dimension (deliciousness) and the indirect inference dimension (healthiness).

### 5.3. Materials

The 18 food names were identical to those in Experiment 1, and 18 food images were created based on these names.

### 5.4. Procedure

In Experiment 2, the 18 combinations of food name and the corresponding images were randomly divided into two 9-trial presentation blocks. Participants were told to view the food name/image combination at their own self-pace and rate the direct perception dimension (deliciousness) and the indirect inference dimension (healthiness) on a 7-point rating scale, which was used in a previous study by Pathak [47].

### 5.5. Results

A two-way repeated-measures ANOVA was conducted using SPSS 24.0 to analyze the rating scores. The Kolmogorov–Smirnov (K-S) test showed that four data sets satisfied the assumption of normality (*p* > 0.05).

The results (Table 3) showed that there was no significant difference between ratings scores of food names with a high concreteness level (*M* = 5.10, *SD* = 0.84) and those with a low concreteness level (*M* = 5.10, *SD* = 0.75), *F* (1,100) < 0.001, *p* = 0.98, *η*^2^ < 0.001. The rating scores of deliciousness (*M* = 5.29, *SD* = 0.73) was significantly higher than the rating scores of healthiness (*M* = 4.91, *SD* = 0.81), *F* (1,100) = 30.89, *p* < 0.001, *η*^2^ = 0.24. The interaction between the concreteness level and the evaluation dimension was significant, *F* (1,100) = 41.04, *p* < 0.001, *η*^2^ = 0.29.

**Figure 2:**
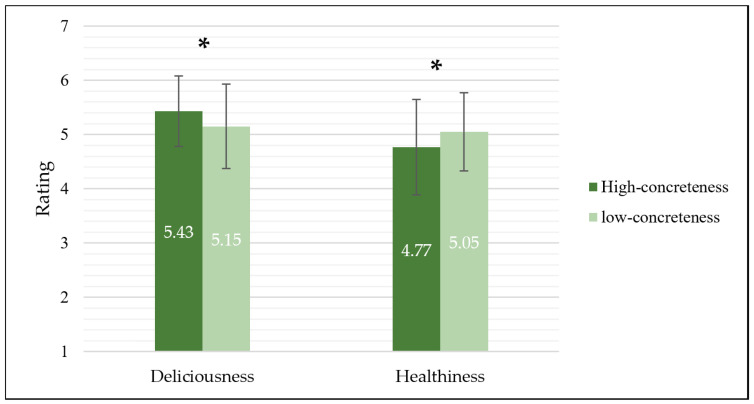
Evaluations of food deliciousness (**left**) and healthiness (**right**) for food names with different levels of concreteness. Error lines represent standard deviations. * represents significant difference.

The simple effects analysis (Figure 2) revealed that the rating scores of deliciousness in the high-concreteness group (*M* = 5.43, *SD* = 0.65) were significantly higher than those in the low-concreteness group (*M* = 5.15, *SD* = 0.78), *F* (1,100) = 20.81, *p* < 0.001, *η*^2^ = 0.17. However, when evaluating healthiness, the rating scores in the high-concreteness group (*M* = 4.77, *SD* = 0.88) were significantly lower than those in the low-concreteness group (*M* = 5.05, *SD* = 0.72), *F* (1,100) = 23.02, *p* < 0.001, *η*^2^ = 0.19.

## 6. Discussion

This study aimed to explore people’s perceptions of food names with different levels of concreteness and their evaluations of food deliciousness and healthiness. Experiment 1 used a subjective guessing paradigm to investigate the likelihood that names with different levels of concreteness would be perceived as foods or dishes. The results showed that compared to low-concreteness names, people were more inclined to judge high-concreteness names that were easier to construct mental images of as foods or dishes, confirming hypothesis one. The reason may be that high-concreteness names often include the food’s ingredients (main ingredients, additional ingredients, seasonings), taste, color, and cooking methods, making it easier for people to form a direct impression and visualize the dish’s appearance, taste, and texture. Additionally, high-concreteness names can convey more information in a short time, allowing consumers to identify and imagine food without extra cognitive effort. This processing is typically smoother and more likely to stimulate people’s sensory and emotional experiences.

Based on hypothesis one, the present study further speculated that consumers’ evaluations of food with high-concreteness names are significantly higher in terms of the direct perception dimension (deliciousness) but lower in terms of the indirect inference dimension (healthiness) than those of foods with low-concreteness names. Therefore, Experiment 2 explored the impact of food names with different levels of concreteness on consumers’ perceptions and evaluations in the direct perception dimension (deliciousness) and the indirect inference dimension (healthiness). The results showed that foods with high-concreteness names were rated significantly higher in terms of deliciousness than foods with low-concreteness names but significantly lower in terms of healthiness than foods with low-concreteness names. The results indicate that compared to foods with low-concreteness names, foods with high-concreteness names are perceived as more delicious but less healthy.

In this experiment, the mean familiarity scores for both groups of food name stimuli were below 4, indicating that both the high- and low-concreteness food names were of low familiarity. This indicated that the interference of familiarity was eliminated and ensured that participants could imagine the food represented by the names rather than simply recalling them [24].

When individuals attempt to understand food names to make decisions or judgments, they first try to visualize the food based on the name and tentatively combine this mental image with the visual information provided [36]. When it is difficult to construct a coherent image based on the provided visual and verbal information, consumers often form unfavorable evaluations of the product. Petrova and Cialdini [48] revealed that when consumers struggle to integrate the mental images evoked by verbal information with the images in advertisements, the effectiveness of the advertisement decreases. Therefore, compared to high-concreteness food names, low-concreteness food names make it more difficult for consumers to construct mental images and combine them with visual information, leading to a lack of coherence between visual and verbal information. This explanation also aligns with the basic principles of the feelings-as-information theory. People tend to process high-concreteness food names more fluently, leading to greater preferences. However, low-concreteness food names, due to their lack of vividness and specificity, make it harder to imagine or understand the food mentally. This leads to processing difficulties, meaning that information is processed less fluently, which can evoke negative experiences. As a result, individuals are more likely to give negative evaluations in the direct perception dimension (deliciousness).

However, Experiment 2 also found that foods with low-concreteness names were rated significantly higher in healthiness than foods with high-concreteness names. Previous research has indicated that people tend to evaluate items with descriptive names more positively, perceiving them as more delicious and higher in calories than similar foods with conventional names [16]. This effect may be due to the fact that high-concreteness food names contain more descriptive contents and provide more clues about the food. In contrast, low-concreteness food names make it more difficult for individuals to understand the ingredients and cooking methods and to assess the caloric content, making these foods more likely to be judged as healthy than those with high-concreteness names. Additionally, considering that people often believe in an inverse relationship between seriousness/usefulness and fun/enjoyment, there is a similar relationship between healthy and delicious foods [40,49]. Therefore, foods with high-concreteness names are perceived as more delicious but less healthy.

In summary, the existing research on food marketing explored the impact of food names on consumer attitudes and behaviors [18,50], yet there is limited research on the effects of food names with different levels of concreteness. With the rising trend of naming foods with abstract names, it is crucial to investigate the influence of levels of concreteness in food names on consumers’ perceptions of food deliciousness and healthiness. This study explored how the concreteness level of food names affects consumers’ perceptions of food deliciousness and healthiness. Theoretically, there has been a significant debate in previous research regarding the effects of the abstractness and concreteness of information, and this debate also exists in marketing and consumer psychology. This study addresses this debate, to some extent, by distinguishing between two evaluation dimensions—direct sensory dimensions and indirect inference dimensions. Our findings indicate that high-concreteness food names have an advantage in the direct sensory dimension (deliciousness), while low-concreteness food names are perceived as healthier in the indirect inference dimension (healthiness). Practically, this finding offers a strategy for restaurant operators and food service providers to use high-concreteness names for dishes, providing consumers with more direct information, enhancing processing fluency, and leading to more favorable evaluations of deliciousness. On the other hand, low-concreteness food names may lead consumers to perceive these foods as healthier. Current global consumer trends indicate that people are placing increasing emphasis on health [51] and recognizing the benefits of healthy foods [49]. Therefore, the restaurant industry may effectively emphasize the health attributes of food in marketing by using low-concreteness food names to describe food. This approach can guide consumers to engage in more cognitive processing, thus increasing their evaluations of the healthiness of food. Additionally, policymakers and health promoters can leverage these insights to increase the consumption of healthy foods.

Future research should further explore the following areas. First, Cardello [52] found that different foods elicit different emotional responses, and these emotional responses are related to people’s food preferences. Studies have shown that people in positive emotional states tend to consume healthy foods, while negative emotions are associated with a tendency to consume junk food [53,54,55,56]. Therefore, whether the concreteness of food names can lead to changes in food consumption preferences through emotional experiences is an area that requires further investigation. This could also provide additional explanations for the psychological mechanisms behind the finding that foods with high-concreteness names have an advantage in the direct perception dimension (deliciousness), whereas foods with low-concreteness names have an advantage in the indirect inference dimension (healthiness).

Additionally, Irmak et al. [13] found that changes in food names impact the health and taste evaluations, as well as consumption ratings, of dieters and nondieters. Future research should include more variables such as emotional levels and whether the participants are dieters.

Furthermore, Yang et al. [57] discovered that language cognition ability in the general Chinese population follows an inverted U-shaped relationship with age, peaking in the 20–30 age group and gradually declining nonlinearly thereafter. Therefore, from a developmental perspective, whether language cognition ability and food names also interact to affect people’s food consumption preferences is another aspect that future research should consider further.

Finally, this study currently lacks some field research evidence. Future studies should consider incorporating field experiments to improve the ecological validity of the research.

## 7. Conclusions

In summary, this study reaches the following preliminary conclusions:

1. Compared to low-concreteness names, high-concreteness names are more likely to be judged as foods or dishes.
2. Foods with high-concreteness names have an advantage in the direct perception dimension (deliciousness), whereas foods with low-concreteness names have an advantage in the indirect inference dimension (healthiness).

## Figures and Tables

**Table 1:** The means and standard deviations (*SD*) of the concreteness level, familiarity of food names, and deliciousness of food images.

	High-Concreteness	Low-Concreteness
Concreteness level	**4.37 (1.07)**	**2.51 (0.88)**
Familiarity	2.49 (0.81)	2.19 (0.72)
Deliciousness	5.16 (0.60)	4.93 (0.85)

Bolded indicates statistically significant.

**Table 2:** Paired-sample *t*-test in Experiment 1.

Variable		*df*	*t*	*p*	Cohen’ *d*
Pair	High-concreteness	101	11.09	<0.001	1.10
	Low-concreteness				

**Table 3:** Repeated-measures ANOVA in Experiment 2.

Variable	Factors	*F*	*p*	*η^2^*
Rating	Concreteness level	<0.001	0.98	<0.001
	Evaluation dimension	30.89	<0.001	0.24
	Concreteness level × Evaluation dimension	41.04	<0.001	0.29

## Data Availability

The original contributions presented in the study are included in the article, further inquiries can be directed to the corresponding authors.
